# Arterial Stiffness and Renal Replacement Therapy: A Controversial Topic

**DOI:** 10.1155/2015/729609

**Published:** 2015-05-07

**Authors:** Edmundo Cabrera Fischer, Yanina Zócalo, Cintia Galli, Sandra Wray, Daniel Bia

**Affiliations:** ^1^Favaloro University (AIDUF-CONICET), Solís 453, C1078AAI Buenos Aires, Argentina; ^2^Technological National University, C1179AAQ Buenos Aires, Argentina; ^3^Physiology Department, School of Medicine, CUiiDARTE, Republic University, 11800 Montevideo, Uruguay

## Abstract

The increase of arterial stiffness has been to have a significant impact on predicting mortality in end-stage renal disease patients. Pulse wave velocity (PWV) is a noninvasive, reliable parameter of regional arterial stiffness that integrates the vascular geometry and arterial wall intrinsic elasticity and is capable of predicting cardiovascular mortality in this patient population. Nevertheless, reports on PWV in dialyzed patients are contradictory and sometimes inconsistent: some reports claim the arterial wall stiffness increases (i.e., PWV increase), others claim that it is reduced, and some even state that it augments in the aorta while it simultaneously decreases in the brachial artery pathway. The purpose of this study was to analyze the literature in which longitudinal or transversal studies were performed in hemodialysis and/or peritoneal dialysis patients, in order to characterize arterial stiffness and the responsiveness to renal replacement therapy.

## 1. Introduction

Hemodialyzed and peritoneal dialysis patients are currently evaluated in order to detect structural and functional alterations in the arterial tree. Since it has been demonstrated that renal function replacement therapy significantly improves survival rates of end-stage renal disease (ESRD) patients, the improvement of biochemical and hemodynamic parameters is desirable aims. Nevertheless, the underlying mechanism by which these improvements occur is not entirely understood, and not all cardiovascular parameters tend to reach their normal values. Pulse wave velocity (PWV) is a well-known independent predictor of cardiovascular disease that is currently obtained by using a simple, noninvasive technique. This method to quantify arterial stiffness has been used during the last decades, and a good number of analyses have been reported, including longitudinal studies. Furthermore, cross-sectional studies were performed, including the comparative analysis between hemodialysis and peritoneal dialysis patients. Nevertheless, the relationship between renal replacement therapy and arterial stiffness remains unclear, since the research results are controversial; sometimes they are inconsistent and other times it is not possible to compare them.

The purpose of this work was to analyze the literature in which longitudinal or transversal studies were performed in hemodialysis and/or peritoneal dialysis patients and (1) to summarize the effects of the dialysis modality on arterial stiffness and (2) to comment on the relationship of arterial stiffness impairment with the etiological factors mentioned in the revised literature. For practical reasons, the text was organized taking into account the dialysis modality and the nature of the research: whether it is a transversal or a longitudinal study. When comparisons among the reported researches were feasible, the results were included in tables.

## 2. Arterial Stiffness and End-Stage Renal Diseases

In recent years, great emphasis has been placed on the role of arterial stiffness in the development of cardiovascular diseases. Aortic stiffness, which results in increased pulse pressure (PP), cardiac overload, and left ventricular hypertrophy, is an established predictor for cardiovascular morbidity and mortality in several disease (i.e., chronic kidney disease, CKD). Indeed, the assessment of arterial stiffness is increasingly used in the clinical assessment of patients.

Basically, arterial stiffness can be measured “systemically” (the cardiovascular system as a whole), “regionally” (large arterial segments or pathways), or locally (arterial rings or short arterial segments) [1, 2]. In contrast to systemic arterial stiffness, which can only be estimated from models of the circulation (i.e., Windkessel model), regional and local arterial stiffness can be measured directly and noninvasively, at various sites along the arterial tree. A major advantage of the regional and local evaluations of arterial stiffness is that they are based on direct measurements of parameters strongly linked to wall stiffness [1, 2]. Local arterial stiffness is directly determined, from the change in pressure driving the change in arterial diameter (distension or volume). However, because it requires a high degree of technical expertise and different techniques (i.e., pressure measurement using applanation tonometry and diameter measurement using ultrasound) and takes longer than measuring “regional stiffness”, local measurement of arterial stiffness is only really indicated for mechanistic analyses in pathophysiology, pharmacology, and therapeutics, rather than for epidemiological or clinical studies [1, 2].

In contrast, the measurement of arterial pulse wave velocity (PWV: a regional stiffness marker) is generally accepted as the most simple, noninvasive, robust, and reproducible method to determine arterial regional stiffness. Measured along the aortic and aortoiliac pathway, it is the most clinically relevant, since the aorta and its first branches are what the left ventricle “sees” and are thus responsible for most of the pathophysiological effects of arterial stiffness. Carotid-femoral PWV has been used in the epidemiological studies demonstrating the predictive value of aortic stiffness for cardiovascular events. In contrast, PWV measured outside the aortic pathway (i.e., at the upper or lower limb) allows determining the arterial stiffness in those regions but has no predictive value. The Moens-Korteweg equation states that PWV is proportional to the square root of the incremental elastic modulus (*E*_inc_) of the vessel wall given constant ratio of wall thickness, *h*, to vessel radius, *r*, and blood density, *ρ*, assuming that that the artery wall is isotropic and experiences isovolumetric change with pulse pressure [1, 2]:

(1) $$\begin{array}{c} \text{PWV}=\sqrt{\frac{E_{\text{inc}}\cdot h}{2r\rho }}. \end{array}$$

Epidemiological studies in almost all populations have clearly shown that aging is the most determinant risk factor for cardiovascular diseases. This age-associated risk for the development of cardiovascular complications is associated with numerous normal or physiological deleterious changes in the structure and function of the arterial system (i.e., increase in aortic PWV) [3]. Among the most characteristic changes associated with aging are central arteries stiffening and remodeling (i.e., intima and/or media layers thickness increase, increased length, and tortuousness) of large arteries. The degeneration of elastic (elastine) fibers is associated with an increase in collagen fibers and ground substance and depositions of calcium. In this context, in end-stage renal disease (ESRD) patients the arterial structural and functional changes are characterized by an accelerated influence of aging [4]. Premature or early vascular aging (EVA syndrome) are observed with progression of CKD and in ESRD. This accelerated aging is associated with outward remodeling of large and medium arteries, characterized by an increased arterial diameter not totally compensated for by artery wall thickness increase. In addition, despite the fact that deposition of calcium salts in the walls of human arteries is a “normal or physiologically” inevitable consequence of aging, the extent of calcifications is more pronounced in ESRD [4]. As will be discussed, arterial stiffening in CKD and ESRD patients is of multifactorial origin.

## 3. Hemodialysis and Arterial Stiffness

### 3.1. Transversal Studies

As was mentioned, the role of vascular calcifications has been pointed out as a determinant factor associated to arterial aging. In 2013, London et al. reported that the incremental elastic modulus (*E*_inc_, i.e., the arterial wall stiffness) of the carotid artery was increased in hemodialyzed patients [4]. This research included 155 hemodialyzed patients whose age (abscissa) versus arterial stiffness (ordinate) relationship (*x*/*y* graph) was higher (a significantly steeper slope) than that obtained in 105 healthy control subjects. Curiously, in the same cohort of hemodialyzed patients, the age versus arterial stiffness relationship of patients without arterial calcifications was lower than that obtained in healthy subjects. This finding is coincident with those reported (by our group) in an animal model of arterial calcinosis [5]. On the contrary, hemodialyzed patients with carotid calcification showed the highest age versus arterial stiffness relationship. Since the age versus arterial stiffness relationship of the noncalcified hemodialyzed patients is lower than that obtained in the healthy cohort, the beneficial role of renal replacement therapy could be ensured. Calcinosis of the media in hemodialysis patients has been linked to increases of arterial stiffness by several authors [6, 7].

On the other hand, an interesting research by Shinohara and coworkers, published a decade ago, demonstrated that hemodialysis has no adverse effects on renal replacement therapy when compared to uremic predialysis patients [8]. The authors emphasized on the active role of the control of metabolic alterations patients have in ESRD (Table 1). The probably most important finding of this research was that hemodialysis patients (*n* = 144) showed significantly lower PWV values (*p* < 0.05) than predialysis patients (*n* = 71).

### 3.2. Longitudinal Studies

In 2001, Guerin and coworkers reported a follow-up (51 ± 38 months) of 150 hemodialyzed patients in which the decrease of PWV values in response to blood pressure declined was associated with a significant improvement of survival rates, which was attributed to the use of ACE inhibitors [9]. The authors divided the patients into blood pressure responders and nonresponders and emphasized on the fact that normalization of mean arterial pressure* per se* was unable to determine a decrease in mortality.

In 2005, Takenaka and Suzuki demonstrated that patients in hemodialysis (*n* = 15) showed a significant increase of PWV in a short period of six months (from 11.82 ± 0.54 m/sec to 14.56 ± 1.20 m/sec; *p* < 0.05). Following the mentioned impairment of the arterial stiffness, the same cohort of patients significantly decreased their PWV values after six months of administration of Sevelamer (13.34 ± 0.90 m/sec). In this small cohort, patients with marked hyperparathyroidism and/or receiving Vitamin D pulse therapy were excluded [10].

An interesting study was carried out by a Canadian group in hemodialyzed patients after a follow-up of 1.2 years [11]. Surprisingly, in this cohort the aortic (carotid-femoral) PWV increased (from 13.27 m/s to 14.26 m/s; *p* < 0.001) (Table 1) and the carotid-radial PWV decreased (from 8.80 m/s to 8.05 m/s; *p* < 0.001) although no convincing argument was provided about this rather strange finding.

A 36-month follow-up of hemodialyzed patients (*n* = 80) and general population patients (*n* = 60) concluded that arterial stiffening was accelerated in hemodialyzed patients. Furthermore, the authors mentioned that cholesterol and uremia-related factors were determinants of the increased arterial stiffness in hemodialyzed patients [12].

Recently, a group from London (United Kingdom) analyzed the relationship of dialysate calcium concentrations on aortic PWV in 289 hemodialysis patients [13]. Arterial stiffness was measured in four subgroups of patients at the beginning of the research and after six months, during which four different calcium concentrations were used. The authors concluded that PWV increased in the whole population (*p* < 0.001), with no changes in the augmentation index (a wave reflection parameter) nor in central (aortic) blood pressure. No significant changes in PWV were found at the start or end of the study in the four subgroups. It was observed that PWV increased in the subgroup of hemodialyzed patients using the lowest dialysate calcium over the six months study. The authors concluded that factors other than the calcium concentration used during hemodialysis are involved in arterial stiffening.

The effects of hemodialysison aortic stiffness have been reported in an old paper by London and coworkers, whose purpose was to study the left ventricular hypertrophy and survival of 153 hemodialyzed patients [14]. The follow-up period was 54 ± 37 months and baseline value of PWV was 11.15 ± 2.70 m/s versus 11.03 ± 3.18 m/s at the end of follow-up, showing a nonsignificant decrease. According to the response in terms of left ventricular hypertrophy, this cohort was divided into responders (decreased by > 10%) and nonresponders (increased or decreased by < 10%); interestingly the responders significantly decreased PWV at the end of the follow-up period (from a baseline value of 11.37 ± 2.80 m/s to 10.18 ± 2.57 m/s; *p* < 0.001). See Table 1.

This distinct response to hemodialysis was not observed in a five-year follow-up of 25 hemodialyzed patients carried out by our group (Argentina and Uruguay), in which a significant decrease of carotid-femoral pulse wave velocity was demonstrated [15].

## 4. Peritoneal Dialysis and Arterial Stiffness

### 4.1. Transversal Studies

Brachial-ankle PWV (a parameter influenced for the aortic and lower-limb arterial stiffness levels) has been used to analyze the correlation between arterial stiffness and several different parameters in a sample of peritoneal dialysis patients in Macao, China [16]. The patients included in the study (*n* = 96) were divided into two groups: (a) low brachial-ankle PWV group including those patients with values below the mean value of the entire population and (b) high brachial-ankle PWV group including those patients with values higher than the mean value of the entire population. The latter were significantly older than the former and included a high proportion of females (*p* < 0.004). The whole population of peritoneal dialysis patients showed that brachial-ankle PWV is independently correlated with age, serum albumin level C-reactive protein, and residual renal creatinine clearance.

### 4.2. Longitudinal Studies

Szeto and coworkers affirmed, in a very simple way, that peritoneal dialysis patients significantly increased carotid-femoral PWV in 24 months [17]. This increase was also observed in carotid-radial PWV. In contrast, Tang and coworkers showed that a PWV improvement found in peritoneal dialysis patients is associated with modifiable risk factors [18]. The authors found that 23% of patients undergo a decrease of carotid-femoral PWV values after a six-month follow-up, compared to the baseline data (Table 2). The outcomes in PWV obtained in these peritoneal dialysis patients were divided into “progressors” (increases higher than 1 m/s or 15% between baselines to 6-month follow-up) and “regressors” (decreases lower than 1 m/s or 15% between baselines to 6-month follow-up).

Coincidentally with the above-mentioned publication of Tang et al., another study in which the cohort outcome of peritoneal dialysis patients was divided was reported by Demirci and coworkers [19]. They analyzed carotid-femoral PWV in 89 peritoneal dialysis patients at baseline and after a nine-month follow-up. Patients with an aortic PWV variation higher than 5% were considered progressors, and, on the contrary, patients with equal or lower values than 5% of variation were considered nonprogressors (Table 2). Patients included in the nonprogressor group showed a significant decrease of carotid-femoral PWV (from 8.56 ± 2.73 m/s in baseline to 7.30 ± 2.02 m/s after 9 months; *p* < 0.0001).

## 5. Hemodialysis versus Peritoneal Dialysis: Arterial Stiffness

### 5.1. Transversal Studies

A polish group reported an interesting research in which peritoneal dialysis and hemodialyzed patients were compared. No statistically significant differences between groups were found [20]. The carotid-femoral PWV of 35 ambulatory patients receiving peritoneal dialysis was 9.9 ± 1.2 m/s, while aortic PWV of hemodialyzed patients was 10.0 ± 1.4 m/s. The authors concluded that both hemodialysis and peritoneal dialysis increase aortic stiffness. The comparison between PWV of nondiabetic and that of diabetic patients showed significant differences (*p* < 0.01) in both peritoneal dialysis and hemodialyzed patients (Table 3).

On the contrary, hemodialysis and peritoneal dialysis have been shown to improve arterial stiffness comparatively with predialysis subjects [21]. This study included ambulatory peritoneal dialysis patients (*n* = 62), hemodialyzed subjects (*n* = 56), a cohort of uremic patients (*n* = 128), and a control group (*n* = 40) of healthy subjects. The comparative analysis included control, peritoneal dialysis, hemodialysis, and nondialyzed patients in ESRD. The authors concluded that dialysis improved arterial stiffness evaluated through PWV and augmentation index (Table 3).

It has been pointed out that the dialysis modality (peritoneal or hemodialysis) may impact arterial stiffness in different ways. In an interesting in vivo and in vitro work of Chung and coworkers, it has been reported that the duration of renal replacement therapy did not correlate with arterial stiffness [22]. The study was performed in patients undergoing living-donor renal transplantation. The most important finding of this work perhaps is the difference between the in vivo and in vitro (measured in the) determination of arterial PWV. No differences in noninvasive PWV were found among nondialysis, peritoneal dialysis, and hemodialysis patients (Table 3). On the contrary, the in vitro determination of PWV showed that peritoneal dialysis and hemodialysis patients exhibit higher values than nondialyzed patients (*p* < 0.01).

### 5.2. Longitudinal Studies

PWV has been found to be higher in hemodialyzed patients than in those on continuous ambulatory peritoneal dialysis after 12 months of renal replacement therapy [23] (Table 3). Curiously, the cohort of patients on peritoneal dialysis exhibit higher serum cholesterol levels.

## 6. The Nature of the Arterial Stiffening

Aortic stiffening has been demonstrated in early stages of kidney disease and, as mentioned above, is increased in both hemodialyzed and peritoneal dialysis patients. Furthermore, it has been pointed out that continuous peritoneal dialysis could contribute to determine the most important increases of arterial stiffness.

Peritoneal-dialysis and hemodialyzed patients with abnormal increases of aortic stiffness showed a direct correlation between PWV and blood pressure (systolic, diastolic, and mean) values [8, 17, 19]. Nevertheless, the lowering of blood pressure levels is not always followed by decreases of aortic stiffness evaluated through PWV measurements [9]. This fact obviously shows the multifactorial nature of the arterial stiffening observed in uremic patients.

In 2010, Gao and coworkers reported a follow-up in 107 peritoneal dialyzed patients in which carotid-femoral and carotid-radial PWV were measured and the fluid overload was determined [24]. The authors pointed out that the resulting mean values of carotid-femoral PWV were lower than those obtained in a similar population in which salt intake was higher.

Recently, a report by Sipahioglu et al. showed that the aortic stiffness index (*β*; a local marker of the ascending aorta stiffness) measured in a cohort of peritoneal dialysis patients showed higher values than that obtained in healthy subjects [25]. Moreover, in this 2-year longitudinal follow-up, 156 patients showed a significant correlation of the *β* index with age (*p* < 0.001) and Kt/V (*p* < 0.023).

In patients with ESRD there is an increase of degenerative processes that affect the structural integrity of the arterial wall, which involve the deposit of abnormal product derived from proteins or lipids [26]. Several reports mentioned that the reduction of AGEs (Advanced Glycation End Products) improves arterial stiffening [6].

Alteration of calcium and phosphate metabolism has been found to be associated with structural and functional changes of the arterial wall. Particularly, the development of mediacalcinosis has been linked to increases of elastic arterial stiffening [7]. This pathological process which involves the extracellular matrix is accompanied by cellular changes of the arterial wall. As previously reported, uremic toxins resulted in the transformation of vascular smooth muscle cells in osteoblast-like cell [27]; considering this fact together with the increase of arterial stiffness in patients in which the dialysate calcium was high [28], the association between vascular calcification and arterial stiffening is clear. Furthermore, carotid-radial PWV was found to be significantly associated with vascular calcification in a study that included three groups: chronic kidney disease in stage 5, hemodialyzed, and peritoneal dialysis patients [29].

Carotid-femoral PWV was found to inversely correlate with serum albumin levels, obtained in a cohort of peritoneal dialysis patients [30]. Additionally, increases in brachial-ankle PWV have been associated with proteinuria and low creatinine clearance in nondialyzed patients participating in a health-check Japanese program [31]. Phosphate levels have been found to be significantly correlated with arterial wall stiffness in uremic patients [22].

Endothelial dysfunction has been observed in uremic patients and their reversibility was reported in hemodialyzed patients [32]. Hemodialysis has no acute effects on endothelial function and aortic PWV in patients who are on continuous renal replacement function therapy [33].

Arterial stiffness has demonstrated to be correlated with C-reactive protein serum levels [4, 12, 16]. Very few authors performed carotid-radial PWV measurements in patients under renal replacement therapy (Table 4). The reported findings are sometimes inconsistent. For instance, Utescu and coworkers concluded that after a 1.2-year follow-up hemodialysis determined a significant decrease of PWV in upper limbs (arterial stiffness reduction) simultaneous to aortic stiffness significant increases [11]. On the other hand, Szeto and coworkers demonstrated significant increases of carotid-radial PWV after a 2-year follow-up of peritoneal dialysis patients (from 10.05 ± 1.44 m/s to 10.80 ± 1.90 m/s) [17]. Mourad and coworkers concluded that radial arteries showed an increase of arterial stiffness (evaluated through *E*_inc_) in hemodialyzed patients, compared to hypertensive and normotensive control subjects [34]. See Table 4. According to a previous research reported by our group in hemodialysis patients, a significative reduction in the carotid-brachial PWV was demonstrated in the arm where the arteriovenous fistula was confectioned [35]. Moreover, this reduction was greater in patients with the arteriovenous fistula performed in the upper arm than in those with the shunt in the forearm [36].

## 7. Final Comments

The outcome examination of the reports in which a follow-up of arterial stiffness in hemodialyzed patients was performed shows differences, perhaps determined by the lack of a unique data collection methodology. So, the effects of renal replacement therapy on arterial stiffness are a controversial topic. The nature of the differences in terms of arterial stiffness outcomes observed among the above-mentioned reports could be attributed to

- differences among pharmacological drug resources used to control comorbidities and metabolic alterations secondary to renal failure in the analyzed population,
- the use of higher dialysate calcium, which would result in impairment of aortic stiffness,
- the presence or absence of calcinosis of the arterial wall, which was found to be associated with changes in vascular stiffness,
- differences in data collection and type of analysis (transversal and or longitudinal studies).

The list of the factors involved in the source of arterial stiffness alterations at present seems to be incomplete. Nevertheless, the therapeutically connotations of the commented findings are very important, since the stiffening process evidences a high risk for both peritoneal dialysis and hemodialyzed patients.

Finally, it is important to mention that several authors demonstrated a positive relationship between PWV and body volume overload parameters [37, 38]. Furthermore, the arterial stiffness changes could be originated in variations in the exchangeable sodium pool involving the arterial wall, as suggested by Hogas and coworkers [39]. Consequently, it is hypothetically possible that the origin of the observed discrepancies among the analyzed reports in this review could be explained by tissue hydration changes.

## 8. Conclusions

1. The renal replacement therapy effects on arterial stiffness remain controversial.
2. The origin of the arterial wall stiffening found in hemodialyzed and peritoneal dialysis patients is multifactorial.
3. Arterial stiffness increases and decreases seem to be part of a generalized process that involves territories different from the aortic territory, such as the carotid-radial pathway.
4. Contradictory results in arterial stiffness should be analyzed considering all of the variables involved in the determinants of the vascular dynamic behavior, both in elastic and in muscular arteries.
5. Arterial stiffening of elastic and muscular arteries should be evaluated in order to check preventive and therapeutic options that were capable of stopping the impairment of the arterial wall stiffness in hemodialyzed and peritoneal dialysis patients.

## Figures and Tables

**Table 1:** Arterial (Aortic) stiffness in hemodialyzed patients.

	cfPWV in HD	Longitudinal study	Transversal study
Shinohara et al., 2004 [8]	Decrease (1)		X
Charitaki and Davenport, 2013 [13]	Increase (1)	X	
London et al., 2001^∗^ [14]	Decrease (2)	X	
London et al., 2001^∗∗^ [14]	Increase (2)	X	
Utescu et al., 2013 [11]	Increase (3)	X	

Pulse wave velocity in the carotid-femoral pathway (cfPWV) was measured in hemodialyzed patients (HD).

^∗^Responders: patients having a decrease of left ventricular mass by >10% after follow-up.

^∗∗^Nonresponders: patients having an increase of left ventricular mass or decrease <10% after follow-up.

(1) With respect to predialysis values obtained in uremic patients.

(2) With respect to patients receiving hemodialysis for at least 3 months.

(3) With respect to baseline values obtained in hemodialyzed patients (mean = 2.3 years).

**Table 2:** Arterial (aortic) stiffness in peritoneal dialysis patients.

	cfPWV in PD	Longitudinal study	Transversal study
Demirci et al., 2012^∗^ [19]	Increase (1)	X	
Demirci et al., 2012^∗∗^ [19]	Decrease (1)	X	
Szeto et al., 2012 [17]	Increase (2)	X	
Tang et al., 2012^∗^ [18]	Increase (3)	X	
Tang et al., 2012^∗∗^ [18]	Decrease (1)	X	

Pulse wave velocity in the carotid-femoral pathway (cfPWV) was measured in peritoneal dialysis (PD) patients.

^∗^Progressors: patients having an increase of cfPWV after follow-up.

^∗∗^Regressors: patients having a decrease of cfPWV after follow-up.

(1) With respect to baseline values obtained in peritoneal dialysis patients for at least 6 months.

(2) With respect to baseline values obtained in peritoneal dialysis patients for at least 6 months.

(3) With respect to baseline values obtained in the first week of PD training.

**Table 3:** Arterial (aortic) stiffness and modality of dialysis.

	cfPWV in PD	cfPWV in HD	Longitudinal study	Transversal study
Mimura et al., 2005 [23]	Decrease (1)	Increase (1)	X	
Yang et al., 2011 [21]	Decrease (2)	Decrease (2)		X
Strózecki et al., 2012 [20]	Increase (3)	Increase (3)		X
Chung et al., 2010 [22]	Increase (4)	pNS		X

Pulse wave velocity in the carotid-femoral pathway (cfPWV) was measured in peritoneal dialysis (PD) and hemodialyzed patients (HD).

(1) With respect to baseline values obtained in dialyzed patients between 3 and 5 months.

(2) With respect to predialysis values obtained in uremic patients.

(3) With respect to the theoretical value according to Blacher equation.

(4) With respect to nondialysis uremic patients (in vitro studies in humans).

**Table 4:** Arterial (upper-limb or carotid-radial) stiffness in dialyzed patients.

	PD	HD	Longitudinal study	Transversal study
Utescu et al., 2013 [11]		X (PWV)	Decrease (1)	
Szeto et al., 2012 [17]	X (PWV)		Increase (2)	
Mourad et al., 1997 [34]		X (*E*_inc_)		Increase (3)

Pulse wave velocity (PWV) in the carotid-radial pathway or incremental elastic modulus (*E*_inc_) was measured in peritoneal dialysis (PD) or hemodialyzed patients (HD). X indicates the used dialysis modality.

(1) With respect to baseline values obtained in hemodialyzed patients (mean = 2.3 years).

(2) With respect to baseline values obtained in peritoneal dialysis patients for at least 6 months (*p* < 0.0001).

(3) With respect to values obtained in normal normotensive and hypertensive patients (*p* < 0.05).
